# Exploring the opinions of secondary school students on the strengths and weaknesses of the school dental service in Selangor, Malaysia: a qualitative study

**DOI:** 10.1186/s12903-021-01741-7

**Published:** 2021-08-11

**Authors:** Nazirah Ab Mumin, Zamros Yuzadi Mohd Yusof, Jamaludin Marhazlinda, Unaizah Obaidellah

**Affiliations:** 1 Department of Periodontology and Community Oral Health, Faculty of Dentistry, Universiti Sains Islam Malaysia (USIM), Kuala Lumpur, Malaysia; 2 Department of Community Oral Health and Clinical Prevention, Faculty of Dentistry, Universiti Malaya (UM), Kuala Lumpur, Malaysia; 3 Department of Artificial Intelligence, Faculty of Computer Science & Information Technology, Universiti Malaya (UM), Kuala Lumpur, Malaysia

**Keywords:** School dental service, Oral health education, Focus group

## Abstract

**Background:**

The Malaysian School Dental Service (SDS) was introduced to provide systematic and comprehensive dental care to school students. The service encompasses promotive, preventive, and, curative dental care. This study aimed to undertake a process evaluation of the SDS based on the perspectives of government secondary school students in Selangor, Malaysia.

**Methods:**

The study adopted a qualitative approach to explore the opinions of secondary school students on the SDS implementation in their schools. Data from focus group discussions involving Form Two (14-year-olds) and Form Four (16-year-olds) students from the selected schools were transcribed verbatim and coded using the NVivo software before framework method analysis was conducted.

**Results:**

Among the strengths of the SDS were the convenience for students to undergo annual oral examination and dental treatment without having to visit dental clinics outside the school. The SDS also reduced possible financial burdens resulting from dental treatment costs, especially among students from low-income families. Furthermore, SDS helped to improve oral health awareness. However, the oral health education provided by the SDS personnel was deemed infrequent while the content and method of delivery were perceived to be less interesting. The poor attitude of the SDS personnel was also reported by the students.

**Conclusion:**

The SDS provides effective and affordable dental care to secondary school students. However, the oral health promotion and education activities need to be improved to keep up with the evolving needs of the target audience.

**Supplementary Information:**

The online version contains supplementary material available at 10.1186/s12903-021-01741-7.

## Background

To provide dental care to a large number of children, schools represent the most ideal platform to capture the widest possible population. Therefore, school dental service (SDS) is an optimal way to provide dental care to this age group [1]. Comprehensive evidence in the literature proved that SDS is a cost-effective option to improve dental care access for children from a wide range of socioeconomic backgrounds [2–4]. Globally, many countries such as New Zealand, Australia, Singapore, Hong Kong, and Malaysia provide free dental services to school-going children. Such widespread provision results in a significant reduction of untreated dental decay [4].

In Malaysia, the SDS was first introduced in the 1950s [5]. From 1985 onwards, the SDS was expanded to provide a more comprehensive dental care that encompasses promotive, preventive, and curative services with the aim of producing orally fit children by the time they leave school [5]. All students attending government schools in Malaysia receive free annual dental check-ups and treatment from the age of 7–17 years throughout primary and secondary school education. Parental consent for dental treatment is obtained at the beginning of each school year. The promotive component of SDS focused on oral health education (OHE) that is delivered once a year in the respective school by the visiting dental team. As for the preventive component, it includes fluoride varnish application [20,000 parts per million (ppm) fluoride] and fissure sealants for children with high caries risk. Lastly, the curative component includes annual dental check-ups, scaling and polishing, as well as simple restorations of decayed teeth.

To date, most of the evaluations on the SDS in Malaysia involved the assessment of the treatment needs, the types of treatment provided, and the coverage of the services [3]. In 2018, the percentage coverage of primary and secondary schools was as high as 99.3% and 87.5% respectively in the whole country. However, despite the high coverage of SDS, not much improvement was seen in terms of the gingival health among the students. Based on the National Oral Health Survey of School Children 2007 (NOHSS 2007), only 19.6% of 12-year-olds and 10.6% of 16-year-olds presented with healthy periodontium. The caries prevalence for both age groups were 41.5% and 59.6% respectively [6, 7]. Even though the prevalence of caries showed a declining trend from 41.5% in 2007 to 33.3% in 2017, almost all the 12-year-old children (98.8%) involved in the survey had gingival bleeding and required oral hygiene instructions [8]. Additionally, the overall need for preventive care to arrest dental caries also doubled from 11.5% in 2007 to 22.9% in 2017 [8]. Poor oral hygiene and gingival health could be attributed to poor oral health behaviour among the school-going children [9]. Therefore, the effectiveness of OHE in schools is questionable. The method and content of OHE given to this target group need to be reassessed.

As children attend school routinely, SDS represents the most accessible and cost-effective option in providing oral healthcare to children. However, it is paramount to conduct a regular evaluation of the service. Apart from outcome evaluations, further evaluations on the implementation of the SDS are equally important to assess its overall performance [10]. To date, no process evaluation of the SDS has been carried out. Hence, this study aimed to undertake a process evaluation of the SDS by exploring the opinions of secondary school students. Valuable inputs from the perspective of the target group may provide important insights into the improvement of SDS delivery.

## Methods

### Study design

This study applied a qualitative approach to gain insights into how individuals interpret their experiences and the meaning a phenomenon has on them [11]. Through focus group discussions (FGD), the opinions of secondary school students on the implementation process of the SDS were obtained during their interaction and communication with one another in a group setting [12]. With FGD, ideas can be stimulated from all the participants to be further explored. Furthermore, it also allows the researcher to observe their non-verbal behaviours [13]. Therefore, FGD is an appropriate method to evaluate how a programme is being implemented and to identify the needs of the target group for the purpose of developing more meaningful health programmes [14].

### Development of FGD questions and training of the facilitator

Semi-structured open-ended questions were developed and validated by dental public health experts to address the study objective. A trial FGD among eight 16-year-old students facilitated by an expert-trained researcher (NAM) was conducted to test the semi-structured questions, as well as to provide experience to the moderator and to assess the feasibility of FGD under field conditions. Based on the trial results, the open-ended questions were then modified and finalised (Additional file 1).

### Sampling and recruitment

Selangor is the most populated state in Malaysia with the highest number of government secondary schools in the country. Of the nine districts in Selangor, Petaling District is the most central with well-developed infrastructures.

The study population was secondary school students in the Petaling District. A list of secondary schools in the district was obtained from the Ministry of Education’s website. To ensure the participants represented an equal mix of gender and ethnicity, vernacular schools, single-gender schools, and boarding schools were excluded from the sampling. Three co-educational schools that received the SDS and located in different cities and towns within the district were selected via purposive sampling. In each school, Form Two (14-year-old) and Form Four (16-year-old) students from the main ethnic groups (Malay, Chinese, and Indian) who had received and were involved in the SDS activities were purposively selected from classes of different academic performances.

### Data collection, analysis, and ethics

The FGD was held in a separate room in each respective school. Before the FGD, the students received light refreshments and filled in their demographic information in a standardised form. Next, the facilitator (NAM) introduced herself to the students and vice versa before they completed some short-written activities to break the ice. Students were informed that they were allowed to ask questions and that the discussion would be recorded using an audio recorder. After that, the first open-ended question was posed to the students and they were allowed to give opinions and be involved in the group discussion until no further points were raised about the topic. The process was repeated with the second question. Each FGD lasted for about 60 min. A note-taker was appointed to record the time and important key points from the discussion. The facilitator concluded the discussion by summarising the key points and asking the students if they had any additional points to share before thanking them.

The framework method analysis was employed. It is commonly used to get answers or perspectives on specific issues to help address the problems [15, 16]. First, the recorded voice data were transcribed verbatim. Next, the researcher (NAM) read the transcripts a few times to familiarise herself with the raw data. This was followed by the coding process where initial themes were identified via the free coding technique using the NVivo software. Subsequently, a framework was developed and the coded data were indexed into it. In order to improve data transparency, a Microsoft Excel spreadsheet was used to tabulate the data according to the categories in the framework. The data were then summarised and interpreted. Triangulation of data was performed by involving researchers in the same field (ZYMY) and a different field (UO) to read the coded transcripts with the finalised themes [17, 18]. This was to ensure the themes were agreed by all to be accurately reflecting the students’ perspectives on the questions. Any disagreements between the researchers were discussed to achieve a consensus before the themes were finalised.

## Results

**Fig. 1:**
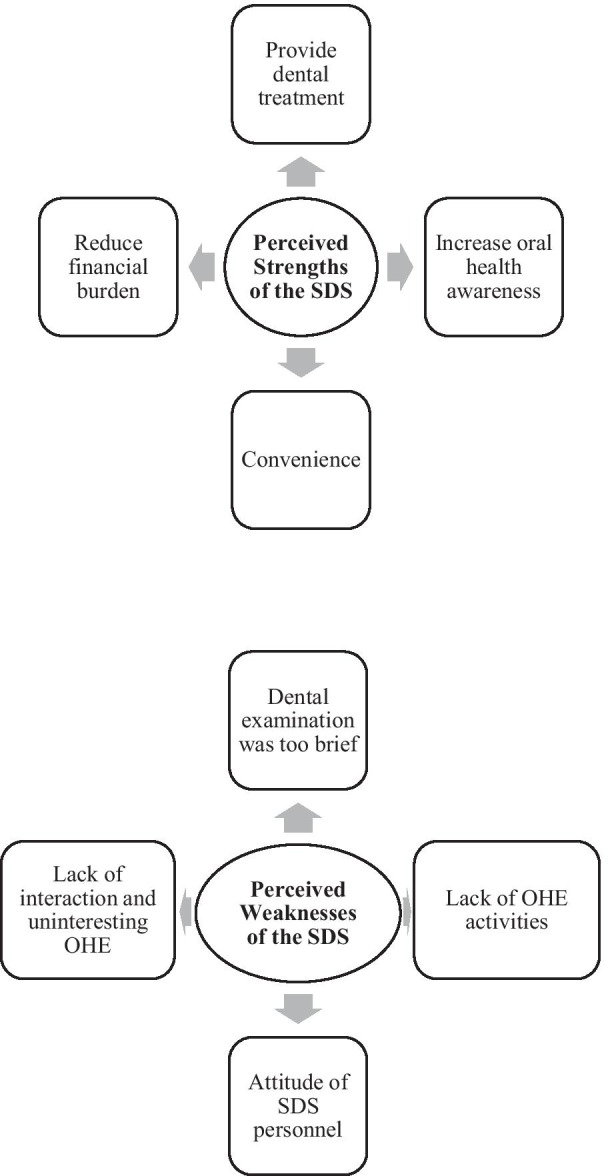
Summary of the students’ opinions on the SDS

A total of 77 students participated in ten FGDs, with 6–8 students per group. Six FGDs consisted of students from upper academic classes and four FGDs consisted of students from lower academic classes. The students’ characteristics are shown in Table 1. Eight themes emerged from the FGD and can be divided into two main headings, namely “Perceived Strengths of the SDS” and “Perceived Weaknesses of the SDS” (Fig. 1).

**Table 1:** Demographic characteristics of the students (N = 77)

Variable	n (%)
Sex	
Male	46 (59.7)
Female	31 (40.3)
Age	
14 years (Form 2)	37 (48.1)
16 years (Form 4)	40 (51.9)
Ethnicity	
Malay	37 (48.1)
Chinese	24 (31.2)
Indian	13 (16.9)
Other (Singh, Punjabi, Iban)	3 (3.9)
Academic performance	
Upper academic class	46 (59.7)
Lower academic class	31 (40.3)

### Perceived Strengths of the SDS

#### Increase oral health awareness

The majority of the students opined that the SDS increased their oral health awareness. As the dental examination at schools is compulsory, all the students must attend the annual dental check-up. They would be informed of the condition of their teeth after the oral examination by the dentist.

If there’s no (dental) check-up, you don’t know what the condition of your teeth is. (Male Student)

...actually, dental check-up at school is good, because from it we can know if our teeth are decayed or not. So we can know and can get them fixed. (Male Student)

Apart from the dental check-up, the information delivered during a dental health talk under the SDS can also improve their awareness about positive habits such as brushing with fluoridated toothpaste and visiting the dentist regularly as well as negative habits including frequent intake of sugary snacks and smoking.

When we are in secondary school at the age of 15 and 16, some may get involved in bad habits like smoking, drinking, etc. These habits may cause bad effects on your teeth and mouth. So, when the dentists talked about oral health and habits, the information given may help them realise that the habits should be avoided or stopped. (Female Student)

#### Provide dental treatment

Most of the students acknowledged that the SDS is important as it provides free dental treatment to students in need, especially those with limited access to dental clinics due to time or financial constraints.

Dental check-up by the dentists is important because if they found a decayed tooth, they will fill it or extract if necessary. (Male Student)

I am happy (with the SDS). It is because if we have any toothache due to cavities, they will put a filling in it. So, the tooth wouldn’t have to be extracted. (Female Student)

#### Reduce financial burden

As the SDS is provided free of charge for all students, many felt that the annual dental check-up is very helpful for students who cannot afford to pay for treatment at a dental clinic. One of the students commented:

The poor can’t afford the money to go to the dentist to check for their teeth and get treatment. (Female Student)

The above statement refers to the visit to a private dental clinic. It is a common understanding among the students that dental treatment from a private dentist can be costly, depending on the type of treatment needed. Therefore, the availability of SDS allows students to receive free dental treatment regardless of their financial circumstances. Another student commented:

My opinion is that the SDS is good. And it eases the poor as well, as they don’t have to fork out money (to get their teeth treated). (Male Student)

The students’ responses showed that they were grateful for the free dental treatment in the SDS and found it to be beneficial, especially for their less affluent friends.

#### Convenience

The students felt that having a dental team that came to the school to provide dental treatment was very convenient for them to get their teeth checked and treated. They did not have to take time off school or miss classes to attend a dental appointment. Also, they need not have to wait long to see the dentist as compared to seeing a dentist in a government dental clinic. One student commented:

Having dental check-ups done at school is convenient. If they don’t do it at school, we have to go to government or private dental clinics outside the school. Also, the waiting time in government clinics is usually long. If we go to private dental clinic, we must pay expensive fees. It is good to have it done in school. Convenient for all students. (Female Student).

### Perceived weaknesses of the SDS

#### Dental examination was too brief

The majority of the students felt that the dental check-up was too quick as if they were in a rush. A few of them said the oral examination was done in haste and not properly executed.

The check-up is too fast. Need (to be) a bit more detailed, more precise. (Male Student)

The dentist is quite alright, but I think they don’t really check, they took the mirror, look inside the mouth and it’s over. (Male Student)

There are a lot of students, they want to do it quick. (Female Student).

Another common response relating to dental examinations at school was that no explanation was given about their teeth by the dentists during or after the check-up. A few students said that they were simply asked to leave the room after the check-up without knowing the status of their teeth or mouth.

They checked (our teeth), then write (in the card) and said, next person. Just like that. (Male Student)

It’s like 30 seconds, then, you are asked to go with no explanation. (Female Student)

If we have problem with our teeth, they didn’t tell us the details or provide any information about what is wrong. They just check and then we have to go. (Female Student)

One of the students even described the dental examination as a silent procedure.

They were very quiet. They didn’t say anything. (Female Student).

However, one student said the dentist did communicate with him briefly.

They call every class for a check-up, if the teeth are not bad, they said you may go. (Male Student).

#### Lack of OHE activities

Many of the students talked about the lack of OHE activities at the school by the SDS team, specifically in relation to dental health talks. The students commented extensively about the dental check-up. In comparison, many commented that dental health talks were seldom conducted by the SDS team and they could only recall the dental health talks they had in the primary schools. A few even commented that there was no dental campaign conducted by the SDS team.

During daily schooling, I never really had a dentist who came to teach us about dental health. (Female Student)

Among all the health talks that we had such as safe sex and all, dental health is basically very rare. It’s almost non-existent. (Female Student)

There’s no (oral health) campaign, I think. So they should start having some campaign. (Male Student)

Some students mentioned they had never been taught how to clean their teeth properly in secondary schools.

“They only check our teeth, but they don’t show us how to brush our teeth correctly. They should show us how to brush our teeth correctly, when to brush, everything.” (Male Student)

Some students said the dentist asked them if they or their parents were smokers and went on to advise them not to smoke. However, the dentist did not provide an explanation as to why smoking is bad for oral health:

Sometimes the dentist asked if we or our parents smoke. But they only asked the question and did not give us any good reasons why we should not smoke. (Female Student).

#### Attitude of SDS personnel

A few students commented that the SDS personnel tend to be less friendly. Some of them were quoted as saying the dentist or dental therapist who examined them appeared to be very serious. One male student said he wished the SDS personnel could be gentler and more soft-spoken.

And they (were) rushing me to sit on the chair, check my teeth and everything. The dentist said “Come now! Sit down now! I want to finish fast. (Male Student)

Some of them admitted that the dentist on duty was not happy if they came to the examination room late. One student commented:

If you come late, they will be crossed at you, I’ve experienced that. Yes, I happened to be late when I was in Form 2 (two years ago) and the nurse scolded me. (Female Student)

#### Lack of interaction and uninteresting OHE

Almost all students agreed that the occasional dental health talks given by the SDS were uninteresting. As a result, they were not interested to listen to the talks unless the dentist made some jokes to help them stay focused.

For me, the talk is boring because I don’t like listening to talks. I prefer interaction. If they make funny jokes, it will be more fun and interesting. (Female Student)

One student commented that the dental health talk was less interesting because the content was repetitive:

Quite boring. Because it’s repeating the same thing we already know. (Male Student).

Apart from that, the students felt that the school hall was an unsuitable venue for dental health talks as the distance to the stage was quite far. Thus, students seated at the back would not be able to pay attention due to the surrounding noise. When asked about their experience in the dental talk given in the school hall, one student quipped, *“I was bored.”* (Male Student).

On a similar note, one of the students suggested the inclusion of interactive activities in the dental health talks so that students can pay attention and be more receptive to the messages delivered.

If there are no activities, the students will make noise in the hall, we can’t hear what the dentist is saying, and so we can’t pay attention (Male Student).

## Discussion

This study explored secondary school students’ opinions on the strengths and weaknesses of the SDS. The majority of students agreed that the SDS has contributed positively towards their oral health. In terms of advantages, the SDS provides free and easy access for them to receive dental check-ups and treatment. Such convenience is especially beneficial for students from low-income families. It also increases their awareness about oral health. However, a large number of the study participants raised unfavourable issues regarding the dental examination procedures, OHE activities, and the attitude of the SDS personnel.

The SDS is one of the endeavours undertaken by the Oral Health Division, Ministry of Health, Malaysia with the aim of offering equitable oral healthcare to all school-going children in government schools [5]. Each district in a state has its own SDS team that consists of dentists, dental therapists, dental surgery assistants, and dental attendants from the government dental clinics. The dentists will provide oral healthcare services to the students in secondary schools while the dental therapists will attend to the primary school students. In addition, other responsibilities shouldered by the SDS teams include the provision of annual dental check-ups, treatment, and dental health talks.

In terms of the effectiveness of the SDS, many published studies have shown that SDS greatly improved the oral healthcare of children [19, 20]. Furthermore, the SDS has been found to be more effective in providing equitable paediatric dental care compared to the private-practice model in the United States [4]. On a similar note, the SDS was also effective in Malaysia as it contributed to a substantial reduction in the caries prevalence among school-going children as reported in the NOHSS [8].

Without a doubt, the SDS successfully cater to the dental need of a wide population of children and adolescents. The SDS provides a platform for students to receive dental check-ups and to learn about the condition of their oral health, especially among adolescents who rarely visit dental clinics [21, 22]. As it is conducted in the school setting, SDS helps to overcome logistic barriers that typically restrict access to dental care [20]. The mandatory annual dental check-up is proven to be beneficial as it increases the students’ awareness of their oral health and their access to dental treatment. Subsequently, preventive treatment can reduce the prevalence of untreated dental decay and further aggravation of dental health.

Secondly, the SDS provides a convenient mean for the students to receive dental treatment without having to miss school. With the SDS, students do not have to go to private or government dental clinics for dental treatment as they can get the treatment in school during school hours. This reduces the possibility of students being absent from school. School absenteeism is commonly higher among students from lower-income groups and their education will be further affected if they need to miss school for dental appointments [23]. In other countries, toothache and its treatment at dental clinics are among the common reasons for students’ absenteeism [24, 25]. According to the UCLA Health Policy Research 2009, school-based dental services should be implemented to reduce the problem of students missing school due to oral health issues. This recommendation indicated the importance of SDS [23].

Furthermore, the SDS was deemed beneficial for students from low-income families. This point was raised by the participants during FGD, thus indicating that they acknowledged and appreciated the service. The SDS provides dental treatment to all students regardless of family income and socio-economic status [2, 3], thus reducing the financial burden of parents, especially those from low-income families. This is vital because children from disadvantaged socioeconomic backgrounds are associated with fewer dental visits, higher caries rates, and unmet treatment needs as they cannot afford the dental treatment costs [23, 26]. As commonly known, dental visits can be quite costly, especially if complex procedures are required. Although a previously published study stated that school-based dental care can create inequality among the affluent and the less affluent students due to differences in health literacy [27], having a nationwide water fluoridation programme that caters to almost all states in the country acts as a safety net which prevent the progression of dental caries at the population level. As for the students who are identified by the SDS with poor oral hygiene and a high risk of developing caries, a targeted clinical prevention will be provided, for instance, fluoride application and fissure sealant. To further address issues of disparity, applying the concept of proportionate universalism, which was coined by Marmot (2010), can be considered where interventions are tailored according to the level of need [28].

Despite the strengths, there are some weaknesses of the SDS. For one, many students felt dissatisfied because the dental examination was done in a hurry with minimal interaction between the SDS personnel and the students. A similar study in Malaysia also highlighted that one of the six main factors that caused dissatisfaction about dental services was that the dental examination was not done thoroughly [29]. This could be attributed to time constraints because of the heavy workload for the SDS team to provide services to all the government schools under their coverage area [30]. With a huge number of students to attend to, the dental team might tend to rush through the examinations to ensure that they can examine every student within the stipulated duration. However, this can result in a compromised dentist-patient relationship, causing the students to feel upset as no explanation was given about their oral health. Similarly, a study by Othman and Razak [3] reported that the students felt most dissatisfied when the dental operators did not provide any explanation before carrying out the treatment. A qualitative study among adolescents in Sweden also revealed a similar finding where the respondents expressed hope for their dentists to provide more information about their oral health after the examination [31].

In addition, one of the themes that repeatedly emerged during the FGD was the lack of OHE activities by the SDS. Most of the secondary school students could only recall OHE from their experiences in primary school. According to some students, OHE activities were seldom conducted. Most of them could not recall the last dental health talk they attended in the school. A study reported that the majority of dental officers knew of their responsibility to deliver dental health talks during school visits. However, the lack of time, heavy workloads, poor communication skills, and the need for further training were cited as the main barriers for them to deliver the talks [30]. In another qualitative study [31], the participating adolescents proposed that OHE in school should be of age-appropriate content and given on a regular basis. This is in line with the observation in which many students in our study raised questions about their oral health and expressed their desires for more oral-health-related information. A similar finding was reported in a Swedish study [31].

Apart from that, the pedagogy of dental health talks in terms of content and method of delivery were described as uninteresting and repetitive. This finding echoed another study that explored the opinions of adolescents regarding school-based OHE [32]. Stakeholders such as health educators and planners should take note of these feedbacks to improve the delivery of dental health talks. OHE activities should be made more interactive and include information that is relevant and important to adolescents [33]. Further research is warranted to better understand adolescents’ views on oral health to customise the appropriate intervention programs for them [34]. It is imperative to take into consideration the perspective of the target population in the efforts to improve the uptake and effectiveness of dental health talks.

Next, the poor attitude of the SDS personnel was also one of the weaknesses cited by the students. Likewise, earlier studies on SDS [3, 29] also reported that many students found SDS personnel to be less approachable as they could be too serious during dental check-ups. The image portrayed by the SDS matters because it will influence the dentist-patient relationships. To build rapport and trust, the students need to have a positive impression towards the SDS personnel. Nevertheless, heavy workload, staff constraint, and high pressure during visits could explain why some SDS personnel appeared to be less friendly.

This study managed to obtain the perspectives of the target group on the SDS using FGD. Having a total of six to eight students in each group was deemed suitable for a meaningful discussion [35]. Although there were some challenges initially, the offering of light refreshments in a casual atmosphere with written activities as ice-breaking sessions proved to be helpful for the purpose [21]. Having students with similar backgrounds in the same group also helped in the discussion. While findings from the FGDs could be considered anecdotal, a saturation point was reached in this study whereby no new ideas emerged in the additional groups. Therefore, the data gained in the study can be seen as the students’ reflections on the SDS provided in Selangor. The findings also provided added value in helping the researchers to understand certain facts that might not have been revealed by quantitative studies. Additionally, the balanced mix of genders and ethnicities among the students ensures that richer data is gained from diverse perspectives within a group setting [12].

Based on the study findings, several recommendations can be made to improve the SDS. Firstly, OHE should be conducted in small groups in a fun and interactive manner to fulfil the target audience’s needs. The conventional one-way approach in dental health talks should be re-evaluated. Personalisation of oral hygiene instructions with prioritisation on prevention is necessary to improve the periodontal status among the students [8]. Secondly, OHE materials should be developed by taking into account the end-users [36]. Considering that many secondary school students nowadays are active users of smartphones, a smartphone application will be more practical as an OHE tool. This study highlighted the scarcity of OHE activities in secondary schools. A concerted effort to overcome this problem by those involved in the planning and implementation of oral health promotion for secondary school students should be emphasised. Perhaps, an alternative method for disseminating oral health information to the students can be considered.

Last but not least, as the staff workload is closely related to their attitude and patient-personnel relationship, it is imperative to look into the workload of the SDS team. Future qualitative research should focus on exploring the perspectives of dentists and dental therapists on the facilitators and barriers of the implementation of the SDS. Such information is vital to improve the welfare of the dental personnel and ultimately, the oral healthcare service received by the students. On the other hand, it should be widely acknowledged that SDS is beneficial in providing curative treatment to school-going children and the service providers must optimise the trust that the recipients put in them so that the best dental outcomes can be obtained. Continuous improvement should be put in place to enable the SDS to keep on providing meaningful and efficient service to the school-going children.

## Conclusion

Based on this research, secondary school students outlined the strengths and weaknesses of the SDS. Despite successfully fulfilling its primary role in providing dental treatment to students, SDS should look into the improvement of OHE by addressing the changing needs of the target group. Considering the findings of this study, the SDS can be further enhanced by making it more relevant to the current generation of school children.

## Supplementary Information

**Additional file 1. **The semi-structured open-ended questions in English and Malay: The semi-structured open-ended questions were used to guide data collection during Focus Group Discussion with participants. Most of the sessions were held using both English and Malay languages interchangeably, unless requested by participants to use only one.

## Data Availability

The main data supporting the findings of this study are included within the article. Further details of the datasets are available on reasonable request by contacting the main author (drnazirah@usim.edu.my).

## Abbreviations

SDS: School Dental Service
OHE: Oral health education
NOHSS: National Oral Health Survey of School Children
