# Supplementary material for: Exploring the opinions of secondary school students on the strengths and weaknesses of the school dental service in Selangor, Malaysia: a qualitative study
Source: BMC Oral Health. 2021 Aug 11;21:394. doi: 10.1186/s12903-021-01741-7 (PMC8359023; doi:10.1186/s12903-021-01741-7)
Supplement: Supplementary file 1 — Additional file 1. The semi-structured open-ended questions in English and Malay: The semi-structured open-ended questions were used to guide data collection during Focus Group Discussion with participants. Most of the sessions were held using both English and Malay languages interchangeably, unless requested by participants to use only one. [file 12903_2021_1741_MOESM1_ESM.pdf]

The Semi-Structured Open-Ended Questions used during Focus Group Discussion (English)

**Introduction**	Hi and Hello everyone,<br>Thank you for participating in today's discussion. I really appreciate you spending some of your time to be here with me. I am Nazirah Ab Mumin, and I will be the moderator throughout this session. I am interested to know about the School Dental Service at your school, which I will refer to onwards as the SDS.<br><br>The focus of our discussion today is about the SDS. You can share with me your experience with the SDS, the activities that they do, including the content and the conduct of their activities. You can give your opinions about the implementation of SDS at your school. There are no right or wrong answers, so you are more than welcome to share your thoughts about things that you like about the SDS, including the things that need to be improved.<br><br>The session will take about 1 hour, depending on how much we talk. Your involvement in today's session is voluntary, you can leave or opt out any time you want or for any reason. I also need to inform you that some of what are said in here will be quoted and shared in my writing. Worry not as it will be totally anonymized, so no one will know who said what.<br><br>I also need to inform that today's session will be audio-recorded. The purpose of this recording is to ensure that I would not miss out any details that form the data for my research. Do not worry, as the recording is solely for the purpose of research, it is confidential and would not be released anywhere else.<br><br>You are allowed to ask any questions if you have any regarding the session, and I am more than happy to clear out any doubts.<br>Before we begin, let's do some ice-breaking activity.

**General question**	1. Can you tell me your experience with the SDS at your school?<br>2. What is your opinion about the implementation of the SDS at your school?<br>Probing questions:<br>- What are the activities done by the SDS when they are at your school?<br>- How do you find the activities done by the SDS?<br>- Can you elaborate on your opinion/experience?
**Question 1**	3. What do you like the most about having the SDS at your school?<br>4. In your opinion, what are the strength/ good points of the SDS in providing dental care at your school?<br>Probing questions:<br>- Can you elaborate on that?<br>- Would you like to explain more?<br>- What do you mean by…?<br>- Can you describe why do you like...?
**Question 2**	5. What are the things that you do not like regarding the SDS?<br>6. In your opinion, what are the weaknesses of the SDS in providing dental care at your school?<br>Probing questions:<br>- Can you elaborate on that?<br>- Would you like to explain more?<br>- What do you mean by…?<br>- Can you describe why do you dislike…...?
**Final question**	7. Is there anything else you want to say or add regarding the SDS at your school?
**Closing remark**	Before we end, I would like to summarise the points that we have discussed today……

	The points that we have gathered today are very important, which I hope will benefit the system to ensure that the SDS will continue to provide efficacious and meaningful oral health programs at all schools.<br><br>I want to thank all of you once again for your participation. Your willingness to be a part of my research is truly appreciated. I have something to give you, a small token of my appreciation for your time. Please fill up this form along with your signature.<br><br>Thank you, and have a good day.

The Semi-Structured Open-Ended Questions used during Focus Group Discussion (Malay)

**Pengenalan**	Selamat sejahtera semua,<br>Terima kasih atas penglibatan kamu semua dalam sesi perbincangan hari ini. Saya sangat menghargai kamu berada di sini. Saya Nazirah Ab Mumin, dan saya akan menjadi pemudahcara sepanjang sesi ini. Saya berminat untuk mengetahui dengan lebih mendalam tentang Perkhidmatan Pergigian di Sekolah, atau nama singkatannya dalam Bahasa Inggeris, SDS.<br><br>Fokus utama perbincangan hari ini ialah berkenaan SDS. Kamu boleh berkongsi pengalaman yang pernah dilalui dengan SDS, aktiviti yang mereka lakukan, termasuk isi kandungan dan cara aktiviti dilaksanakan. Kamu boleh memberi pendapat tentang perlaksanaan SDS di sekolah kamu. Tidak ada jawapan yan gbetul atau salah, jadi kamu sangat dialukan untuk berkongsi perkara-perkara yang kamu suka tentang SDS, termasuk perkara yang perlu ditambahbaik.<br><br>Sesi perbincangan ini akan mengambil masa lebih kurang satu jam, bergantung kepada sebanyak mana isu yang diutarakan. Penglibatan kamu dalam sesi ini ialah sukarela, kamu boleh meninggalkan perbincangan atau menarik diri pada bila masa kamu suka dan untuk apa juga sebab. Saya juga perlu memaklumkan bahawa sebahagian daripada perbincangan hari ini akan dipetikguna dalam penulisan saya. Jangan risau, kerana semua butir peserta akan dirahsiakan, jadi tiada siapa akan tahu siapa penyatanya.<br><br>Saya juga harus memaklumkan bahawa sesi perbicangan akan direkod mengunnakan perakam suara. Tujuan rakaman ini ialah untuk mengelakkan ada maklumat yang tidak direkodkan kerana ini merupakan data kajian saya. Tidak perlu risau kerana rakaman

	ini adalah untuk tujuan kajian semata-mata, ianya bersifat rahsia dan tidak akan disiarkan sebarangan.<br><br>Kamu dibenarkan untuk bertanyakan sebarang soalan sekiranya ada, dan saya akan mencuba sebaik mungkin untuk menjawab agar jelas.<br><br>Sebelum kita mula, mari kita buat sedikit aktiviti untuk berkenalan
**Soalan umum**	1. Boleh kamu ceritakan pengalaman melibatkan SDS di sekolah?<br>2. Apakah pendapat kamu tentang perlaksanaan SDS di sekolah kamu?<br>Soalan menggalak:<br>- Apakah aktiviti yang dijalankan oleh SDS di sekolah kamu?<br>- Bagaimana pendapat kamu tentang aktiviti yang dijalankan oleh SDS?<br>- Boleh kamu ulas dengan lebih lanjut tentang pendapat/pengalaman kamu?
**Soalan 1**	3. Apakah perkara yang kamu gemar/suka tentang perkhidmatan SDS di sekolajh kamu?<br>4. Pada pendapat kamy, apakah kebaikan yang diperoleh daripada SDS dalam memberi perkhidmatan pergigian di sekolah kamu?<br>Soalan menggalak:<br>- Boleh kamu ulas dengan lebih lanjut?<br>- Boleh kamu jelaskan lagi?<br>- Apakah yang kamu maksudkan dengan…?<br>- Bolehkan kamu huraikan kenapa kamu menyukai....?
**Soalan 2**	5. Apakah perkara yang kamu tidak suka/gemar tentang perkhidmatan SDS di sekolah kamu?

	6. Pada pendapat kamu, apakah perkara yang perlu diperbaiki/ kelemahan SDS dalam memberi perkhidmatan pergigian di sekolah kamu?<br>Soalan menggalak:<br>- Boleh kamu ulas dengan lebih lanjut?<br>- Boleh kamu jelaskan lagi?<br>- Apakah yang kamu maksudkan dengan…?<br>- Bolehkan kamu huraikan kenapa kamu tidak nneyukai...?
**Soalan akhir**	7. Adakah perkara-perkara lain yang ingin kamu nyatakan berkaitan perkhidmatan SDS di sekolah kamu?
**Penutup**	Sebelum kita akhiri, saya akan membuat rumusan ringkas tentang isi perbincangan kita hari ini ….<br><br>Segala maklumat yang dikumpulkan hasil daripada perbincangan hari ini adalah sangat penting, yang mana saya harapkan dapat memberi manfaat untuk menambahbaik system dan memastikan SDS akan terus memberi perkhidmatan kesihatan pergigian dengan dengan lebih berkesan dan bermakna di semua sekolah.<br><br>Saya mengucapkan terima kasih sekali lagi di atas penglibatan kamu semua. Kesudian kamu semua melibatkan diri dalam kajian saya amat dihargai. Sebagai tanda penghargaan, saya ingin memberi sedikit saguhati kepada semua yang terlibat. Sila isikan nama dan tandatangan dalam borang ini.<br><br>Sekian , terima kasih.
